# Association between health literacy and patient experience of primary care attributes: A cross-sectional study in Japan

**DOI:** 10.1371/journal.pone.0184565

**Published:** 2017-09-08

**Authors:** Takuya Aoki, Machiko Inoue

**Affiliations:** 1 Department of Healthcare Epidemiology, School of Public Health in the Graduate School of Medicine, Kyoto University, Kyoto, Kyoto, Japan; 2 Department of Family and Community Medicine, Hamamatsu University School of Medicine, Hamamatsu, Shizuoka, Japan; Waseda University, JAPAN

## Abstract

Primary care is regarded as a setting that potentially mitigate patient health literacy (HL) related inequalities. However, there is a lack of evidence about influence of patient HL on the patients’ perception of quality of primary care. We aimed to examine the association between HL and patient experience of primary care attributes. We conducted a cross-sectional survey, and sent questionnaires to adult residents who were randomly selected from a basic resident register in Yugawara Town, Kanagawa, Japan. We assessed HL using a 14-item Health Literacy Scale (HLS-14) and patient experience of primary care attributes using a Japanese version of Primary Care Assessment Tool (JPCAT), which comprises six domains: first contact, longitudinality, coordination, comprehensiveness (services available), comprehensiveness (services provided), and community orientation. We used a multivariable linear regression analyses to adjust individual covariates. Data were analyzed for 381 residents who had a usual source of care. After adjustment for patients’ sociodemographic and health characteristics, patient HL was positively associated with the JPCAT total score (B = 4.49, 95% confidence interval: 0.27 to 8.65 for HLS-14 total score highest quartile, compared with the lowest quartile). Among primary care attributes, HL had significant associations with longitudinality and comprehensiveness (service provided). We found that HL was positively associated with patient experience of primary care attributes in Japanese people. Our findings indicated that greater efforts might be needed to improve patient-centered and tailored primary care to those with low HL.

## Introduction

Health literacy (HL) is the degree to which individuals have capacity to obtain, process, and understand basic health information and services [1]. HL has attracted a lot of attention as a major health issue in clinical care and public health, because various studies revealed that deficiencies in HL was associated with poor health outcomes, negative health behavior, and increased costs [2–6]. Patient HL underpins the efficiency of consultations, health promotion efforts, and self-management programs [5,7,8], therefore it has important applications to clinical care. Especially, primary care is regarded as a meaningful setting to diminish the literacy related inequalities [9]. It is considered that as individual primary care providers should be aware of the frequency with which they see patients with low HL and adjust communication styles to meet the needs of patients and carers, producing better health outcomes and more satisfying encounters for both patients and providers [10]. However, there is a lack of evidence about influence of patient HL on the perception of quality of primary care.

Patient experience is recognized as one of the three pillars of quality in health care, alongside clinical effectiveness and patient safety [11]. Patient experience is considered the most effective measure of patient-centeredness, which is defined as providing care that is respectful of and responsive to patient preferences, needs, and values. This method is increasingly used to assess the quality of primary care, because patient-centeredness is a crucial concept, especially in the primary care setting [12]. A few studies were conducted to investigate the relationship between HL and patient experience of primary care. In these studies, limited HL predicted worse primary care patient experience regarding communication, access, and coordination [13–15]. However, the patient experience measures used in these studies have not been optimized for the primary care setting without sufficient comprehension of core attributes of primary care, which differentiate primary care from other aspects of the health services delivery system [16]. It would be helpful for primary care providers to have a clearer understanding of how HL is related to crucial aspects of patient experience in primary care.

The Primary Care Assessment Tool (PCAT) [17], which was developed by Barbara Starfield and colleagues, is one of the most widely used patient experience measures of primary care attributes. The PCAT permits the assessment of core attributes of first contact, longitudinality, comprehensiveness, coordination, and family and community orientation. We have developed the Japanese version of Primary Care Assessment Tool (JPCAT) and confirmed the validity and reliability of this tool [18].

The key research question of this study was whether people with low HL have worse patient experience of primary care attributes assessed by the JPCAT than people with high HL.

## Materials and methods

We conducted a cross-sectional survey study on a Japanese community-dwelling population to examine the association between HL and patient experience of primary care attributes. The institutional review board of the Hamamatsu University School of Medicine approved this study (approval number E15-089). All study participants provided written informed consent.

### Setting and participants

Potential study participants were randomly selected from residents aged 20–80 years in Yugawara Town in Kanagawa Prefecture, Japan, by using a basic resident register. Yugawara Town is located in the southern part of the Kanto area of eastern Japan. The total population of Yugawara Town was 26,442 according to the 2015 population census [19], with 35.8% of residents ≧65 years old. In terms of occupational structure, employment was provided by primary industry for 3.4%, secondary industry for 17.4%, and tertiary industry for 79.1%.

Of the potential participants, eligible participants were residents who had at least one usual source of care (USC). For this study, we used the same three questions and the algorithm in the JPCAT [18] as an original PCAT adult expanded version [17] to identify an individual’s USC and the strength of that affiliation: (1) Is there a doctor that you usually go if you are sick or need advice about your health? (usual source); (2) Is there a doctor that knows you best as a person? (knows best); and (3) Is there a doctor that is most responsible for your health care? (most responsible). A participant was considered to have a USC if he or she answered positively to any one of the three questions.

We sent a self-administered questionnaire to total 2,000 randomly selected residents from the register of sampling site. The data were collected using a mail survey between June and August 2015. Four weeks after the initial mailing, a reminder was sent out to increase the response rate. Regardless of whether the participants responded to the survey, they were given small gifts worth 200 JPY.

### Measures

#### Health literacy

We used the 14-item health literacy scale (HLS-14) [20] to evaluate HL among participants (S1 File). The HLS-14 is widely used for screening and researches of HL in Japan. This tool assesses all three levels of HL proposed by Nutbeam: (1) functional literacy—sufficient basic skills in reading and writing to be able to function effectively in everyday situations; (2) communicative literacy—more advanced skills to participate actively in everyday activities, to extract information and derive meaning from different forms of communication, and to apply new information to changing circumstances; (3) critical literacy—more advanced skills to analyze information critically and to use this information to exert greater control over life events and situations [21]. The HLS-14 consists of 5 items for functional HL, 5 items for communicative HL, and 4 items for critical HL. The scores on the items (five-point Likert scale) are summed up for each respondent to give the HLS-14 total score, as well as functional, communicative, and critical HL scores. Thus, the HLS-14 total score ranges from 14 to 70 points, with higher scores indicating better HL. The reliability and validity of the HLS-14 were assessed in previous study in Japan [20]. In this study, HLS-14 scores were categorized into quartiles to account for potential nonlinearity.

#### Patient experience of primary care attributes

In this study, we used JPCAT [18] for data collection (S2 File). JPCAT was based on PCAT-AE [17] to measure patient experience of primary care attributes in Japan. This 29-item tool comprises six domains representing primary care attributes: first contact, longitudinality, coordination, comprehensiveness (services available), comprehensiveness (services provided), and community orientation. The scoring system of the JPCAT is structured as follows: each response on a five-point Likert scale is reduced by a factor of 1 and multiplied by 25. The score for each of the domains is computed as the mean value for all converted scale scores in that domain. Thus, the domain scores range from 0 to 100 points, with higher scores indicating better performance. The total score is the mean of the six domain scores and reflect an overall measure of patient experience regarding core primary care attributes. Previous work has shown that JPCAT has good reliability and validity [18]. The primary outcome measure in this study was the JPCAT total score and the secondary outcome measures were the JPCAT domain scores.

#### Covariates

Covariates were selected on the basis of the literature review to identify factors may confound the association between HL and patient experience of primary care. We included covariates for age, sex, years of education, annual household income, self-rated health, and number of comorbidities. All covariates were evaluated as categorical variables by a self-administered questionnaire. Number of comorbidities was assessed by simple counts of disease in each individual, and categorized into three groups: 0, 1, and ≧2.

### Statistical analysis

Descriptive statistics for categorical data are reported as frequencies and percentages; continuous data are reported as means and standard deviation. To determine whether the HLS-14 scores were associated with patient experience of primary care measured by the JPCAT, we used multivariable linear regression analyses. The following possible confounders were included in the analyses: age, sex, years of education, annual household income, self-rated health, and number of comorbidities. We also performed analyses of secondary outcomes to investigate the associations between the HLS-14 total score and each JPCAT domain score using the same linear regression models. For each analysis, we evaluated the null hypothesis with a two-sided significance level of 0.05. For references, a 3-point increase in patient experience measures on a 0–100 scale has been associated with better patient adherence, thus a difference of >3-point is considered significant in magnitude with regard to practical importance [22–24]. To allow for uncertainty in the missing values for independent and dependent variables, we used multiple imputations by fully conditional specification, with the HLS-14 scores, age, sex, years of education, annual household income, self-rated health, number of comorbidities, and the JPCAT scores as variables in the imputation model, thus creating 5 imputed datasets.

According to the sample size formula shown in a previous study, sample size per independent variable values of ≥ 20 were necessary for linear regression analysis [25]. We estimated a minimum sample size of 140 because the maximum number of independent variables was seven in this study. We used R version 3.3.2 (R Foundation for Statistical Computing, Vienna, Austria; www.R-project.org/) and mice package for statistical analyses.

## Results

**Fig 1:**
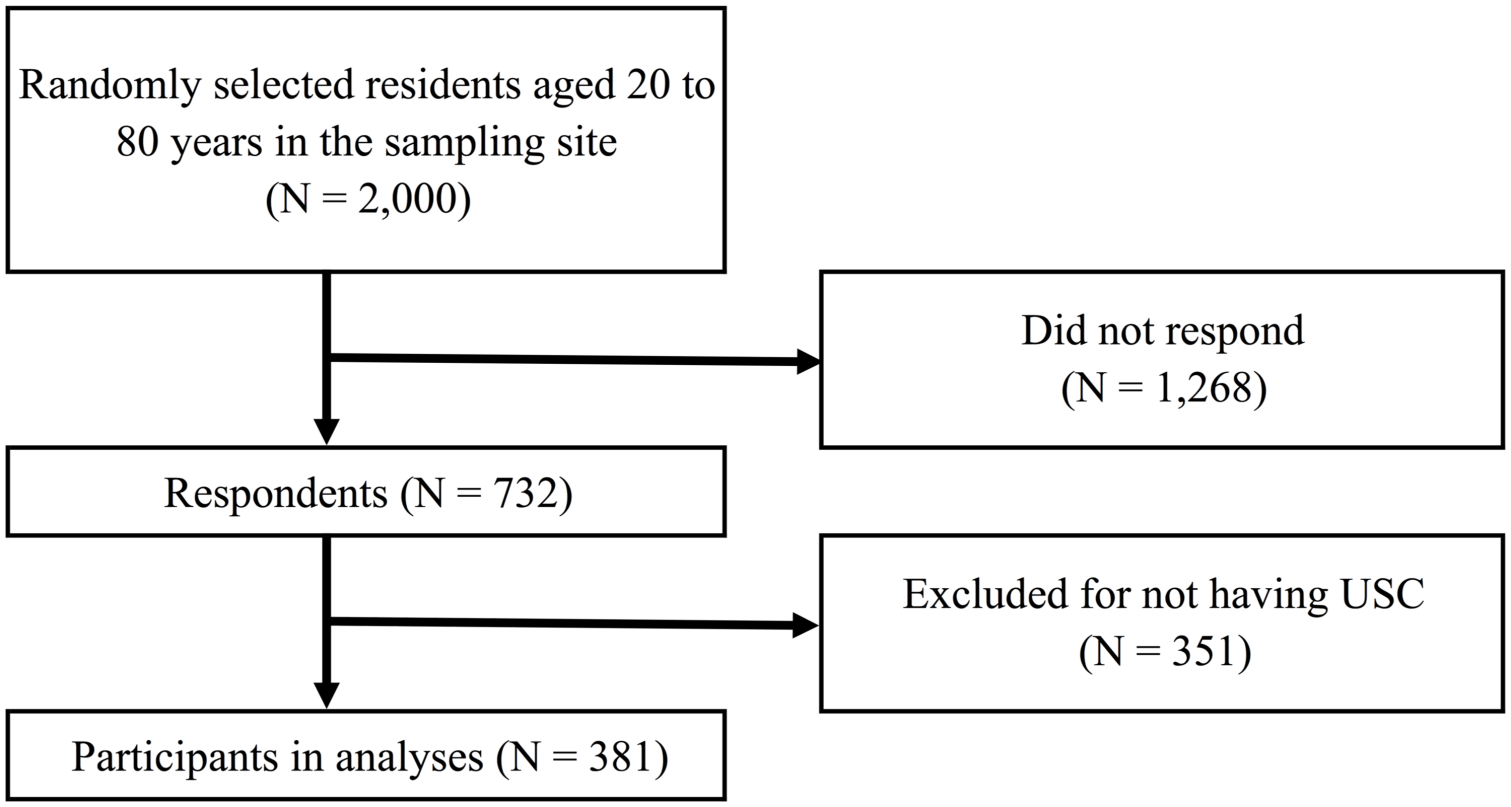
Participant flow chart. USC, Usual Source of Care.

A total of 732 (36.6%) individuals responded to the mail survey. In the responses, we excluded 351 participants who did not have a USC and performed analyses of the 381 eligible participants (Fig 1).

Table 1 shows the distribution of individual characteristics of the 381 eligible participants. The majority of participants were women (64.8%), aged 65 years or above (58.3%). The proportion of participants with multimorbidity was 42.8%. Table 1 also shows the mean and standard deviation of HLS-14 and JPCAT scores. The average HLS-14 total score was 51.2 out of 70 points. The average JPCAT total score was 53.8 out of 100 points; the most highly scored domain was longitudinality (67.7), and the most poorly scored domain was comprehensiveness (services provided) (39.2).

**Table 1:** Participants' characteristics (N = 381).

Characteristic	
Gender [N (%)]	
Male	131 (34.4)
Female	247 (64.8)
Data missing	3
Age (year) [N (%)]	
21–49	52 (13.6)
50–64	100 (26.2)
65–74	161 (42.3)
≧75	61 (16.0)
Data missing	7
Education [N (%)]	
Less than high school	59 (15.5)
High school	163 (42.8)
Junior college	81 (21.3)
More than or equal to college	75 (19.7)
Data missing	3
Annual household income (million JPY) [N (%)]	
<2.00 (≒18,000 US dollar)	89 (23.4)
2.00–4.99	193 (50.7)
≧5.00	78 (20.5)
Data missing	21
Self-rated health [N (%)]	
Very good	26 (6.8)
Good	75 (19.7)
Neutral	150 (39.4)
Poor	85 (22.3)
Very poor	44 (11.5)
Data missing	1
Number of comorbidities [N (%)]	
0	70 (18.4)
1	142 (37.3)
≧2	163 (42.8)
Data missing	6
HLS-14 [mean (SD)]	
Total score	51.2 (7.9)
Functional HL	20.2 (4.2)
Communicative HL	18.0 (3.9)
Critical HL	13.1 (3.4)
JPCAT [mean (SD)]	
Total score	53.8 (15.0)
First contact	48.5 (25.2)
Longitudinality	67.7 (19.1)
Coordination	59.6 (25.2)
Comprehensiveness (services available)	57.8 (23.7)
Comprehensiveness (services provided)	39.2 (25.4)
Community orientation	49.5 (20.7)

HLS-14, 14-item Health Literacy Scale; HL, health literacy; JPCAT, Japanese version of Primary Care Assessment Tool.

Table 2 shows the results of multivariable linear regression analyses modeling the association between the HLS-14 scores and the JPCAT total score as an overall measure of patient experience of primary care attributes. The HLS-14 total score was positively associated with the JPCAT total score, adjusting for age, sex, years of education, annual household income, self-rated health, and number of comorbidities [B = 3.54, 95% confidence interval (CI): -0.92 to 7.99 for HLS-14 total score 2nd quartile; B = 4.16, 95%CI: -0.05 to 8.37 for 3rd quartile; B = 4.49, 95%CI: 0.27 to 8.65 for the highest quartile, compared with the lowest quartile]. In addition, communicative and critical HL scores were positively associated with the JPCAT total score (B = 5.16, 95%CI: 0.70 to 9.63 for communicative HL score highest quartile, B = 4.52, 95%CI: 0.28 to 8.76 for critical HL score highest quartile, compared with the lowest quartile).

**Table 2:** Factors associated with patient experience of primary care attributes (N = 381)^a^.

Scale	B (95% CI)	*P*-value
HLS-14 score (quartile)		
Total		
1st	Reference	
2nd	3.54 (-0.92 to 7.99)	0.123
3rd	4.16 (-0.05 to 8.37)	0.054
4th	4.49 (0.27 to 8.65)	0.038
Functional HL		
1st	Reference	
2nd	0.50 (-3.73 to 4.72)	0.818
3rd	2.91 (-1.94 to 7.75)	0.242
4th	3.31 (-1.38 to 8.00)	0.168
Communicative HL		
1st	Reference	
2nd	0.65 (-3.37 to 4.66)	0.753
3rd	-0.02 (-4.69 to 4.64)	0.993
4th	5.16 (0.70 to 9.63)	0.025
Critical HL		
1st	Reference	
2nd	-0.04 (-4.89 to 4.80)	0.986
3rd	1.81 (-2.02 to 4.80)	0.356
4th	4.52 (0.28 to 8.76)	0.039

HLS-14, 14-item Health Literacy Scale; HL, health literacy; B, unstandardized coefficient.

^a^Patient experience of primary care attributes was measured by Japanese version of Primary Care Assessment Tool. Adjusted for age, sex, years of education, annual household income, self-rated health, and number of comorbidities.

Table 3 shows the results of multivariable linear regression analyses modeling the association between the HLS-14 total score and the JPCAT domain scores. Among primary care attributes assessed by the JPCAT domains, the HLS-14 total score was significantly associated with longitudinality and comprehensiveness (service provided) scores.

**Table 3:** Associations of health literacy with JPCAT domain scores [unstandardized coefficient (95% CI)] (N = 381)^a^.

	JPCAT domain scores					
	First contact	Longitudinality	Coordination	Comprehensiveness (services available)	Comprehensiveness (services provided)	Community orientation
HLS-14 total score (quartile)						
1st	Reference	Reference	Reference	Reference	Reference	Reference
2nd	1.94 (-5.79 to 9.67)	2.60 (-2.67 to 7.87)	0.47 (-6.77 to 7.71)	6.41 (-0.98 to 13.81)	5.46 (-1.86 to 12.77)	4.99 (-1.42 to 11.41)
3rd	-3.34 (-11.16 to 4.48)	4.01 (-1.67 to 9.70)	5.02 (-2.22 to 12.26)	7.49 (0.18 to 14.80)	6.67 (-0.83 to 14.16)	6.92 (1.13 to 12.70)
4th	1.25 (-6.29 to 8.79)	5.70 (0.05 to 11.34)	2.92 (-4.33 to 10.17)	5.53 (-1.60 to 12.67)	10.23 (2.64 to 17.82)	2.48 (-3.60 to 8.56)

HLS-14, 14-item Health Literacy Scale; JPCAT, Japanese version of Primary Care Assessment Tool.

^a^Adjusted for age, sex, years of education, annual household income, self-rated health, and number of comorbidities

## Discussion

Our results revealed that HL was positively associated with patient experience of primary care attributes, especially regarding longitudinality and comprehensiveness, among Japanese people who had a usual source of care. Furthermore, among the levels of HL, communicative and critical HL were associated with patient experience of primary care. According to previous studies, a JPCAT total score difference between the highest HLS-14 total score quartile and the lowest quartile observed in our study was considered significant in magnitude with regard to practical importance.

Although there are previous studies reporting the association between HL and patient experience of access and coordination [13], it had been unclear whether HL is associated with comprehensive patient experience measure of primary care. Our findings indicated that patient HL has a meaningful impact on the patients’ perception of overall quality of primary care, and greater efforts might be needed to improve patient-centered and tailored primary care to those with low HL.

We could make several hypotheses about the mechanisms of how HL influence patient experience of primary care from the results of this study. For example, in this study, HL was positively associated with the longitudinality domain, which mainly evaluates interpersonal continuity [26]. In concordance with this finding, previous studies showed the significance of HL for effective patient-doctor communication [15,27]. As another example, HL was also associated with the comprehensiveness (service provided) domain evaluating the scope of health education services such as physical activity, health information use, and healthy work life balance. According to this result, patients who have limited HL may tend not to understand or remember the content of health education by their primary care provider.

In contrast to the associations of communicative and critical HL with patient experience of primary care, the association between functional HL and patient experience of primary care was not statistically significant. This result might be caused by the characteristics of study population. In countries like Japan, where the basic literacy rate is estimated to be 99% [28], functional HL is considered to play a weaker role than communicative and critical HL in the relationship between HL and understanding of chronic disease care [29]. In concordance with this finding, functional HL might less affect the quality of primary care services from the patient perspective in a population with relatively high functional HL, as in our participants.

The results of this study presented additional findings on the association between HL and patient experience. However, our study had several potential limitations. First, there was a concern about the low response rate, which might potentially introduce selective non-response bias. As we used a self-administered questionnaire, there is a possibility that the patients with very low HL could not participate in this survey. Second, the data were cross-sectional and a causal relationship between HL and patient experience of primary care cannot be definitely established. There is a possibility that better perceptions of quality of primary care attributes lead to improvement in HL. For example, the scope of comprehensiveness of primary care includes health promotion such as education of health information use, which may improve patients’ communicative and critical HL [17,18,30]. However, such relationship should be confirmed in future prospective studies. Third, we did not consider the clustering within practices in the analyses. However, the impact of clustering on the results could be limited, because there are more than 20 different primary care practices in the sampling site. Fourth, the study population covered only a relatively rural town in Japan, where people are mostly literate. In addition, our participants were limited to patients having a USC and had a higher proportion of elderly people and women compared with the Japanese general population. Thus, caution should be taken when generalizing the results of this study.

## Conclusions

We found that HL was positively associated with patient experience of primary care attributes in Japanese people. Our findings indicated that greater efforts might be needed to improve patient-centered and tailored primary care to those with low HL.

## Supporting information

S1 File Item contents of the 14-item health literacy scale. (PDF)

S2 File Item contents of the Japanese version of Primary Care Assessment Tool. (PDF)

S3 File Raw_data.xisk. (XLSX)
