# Supplementary material for: Association between health literacy and patient experience of primary care attributes: A cross-sectional study in Japan
Source: PLoS One. 2017 Sep 8;12(9):e0184565. doi: 10.1371/journal.pone.0184565 (PMC5590975; doi:10.1371/journal.pone.0184565)
Supplement: S1 File — (PDF) [file pone.0184565.s001.pdf]

1. When you read instructions or leaflets from hospitals or pharmacies, how do you agree or disagree about the following?
病院や薬局からもらう説明書やパンフレットなどを読む際に、下記の項目について、あなたはどのように考えていますか？

| | Strongly disagree 全くそう思わない | Disagree あまりそう思わない | Not sure どちらでもない | Agree まあそう思う | Strongly agree 強くそう思う |
|---|---|---|---|---|---|
| Q1 I find characters that I cannot read<br>読めない漢字がある | 5 | 4 | 3 | 2 | 1 |
| Q2 The print is too small for me (even though I wear glasses)<br>字が細かくて読みにくい（メガネなどをかけた状態でも） | 5 | 4 | 3 | 2 | 1 |
| Q3 The content is too difficult for me<br>内容が難しくて分かりにくい | 5 | 4 | 3 | 2 | 1 |
| Q4 It takes a long time to read them<br>読むのに時間がかかる | 5 | 4 | 3 | 2 | 1 |
| Q5 I need someone to help me read them<br>誰かに代わりに読んでもらうことがある | 5 | 4 | 3 | 2 | 1 |

2. If you are diagnosed as having a disease and you have little information about the disease and its treatment, how do you agree or disagree about the following?
ある病気と診断されてから、その病気やその治療･健康法に関することについて、下記の項目について、あなたはどのように考えていますか？

| | Strongly disagree 全くそう思わない | Disagree あまりそう思わない | Not sure どちらでもない | Agree まあそう思う | Strongly agree 強くそう思う |
|---|---|---|---|---|---|
| Q6 I collect information from various sources<br>いろいろなところから知識や情報を集めた | 1 | 2 | 3 | 4 | 5 |
| Q7 I extract the information I want<br>たくさんある知識や情報から、自分の求めるものを選び出した | 1 | 2 | 3 | 4 | 5 |
| Q8 I understand the obtained information<br>自分が見聞きした知識や情報を、理解できた | 1 | 2 | 3 | 4 | 5 |
| Q9 I tell my opinion about my illness to my doctor, family, or friends<br>病気についての自分の意見や考えを、医師や身近な人に伝えた | 1 | 2 | 3 | 4 | 5 |
| Q10 I apply the obtained information to my daily life<br>見聞きした知識や情報をもとに、実際に生活を変えてみた | 1 | 2 | 3 | 4 | 5 |

3. If you are diagnosed as having a disease and you can obtain information about the disease and its treatment, how do you agree or disagree about the following?
ある病気と診断されてから、その病気やその治療･健康法に関することで、自分で見聞きした知識や情報について、下記の項目について、あなたはどのように考えていますか？

| | Strongly disagree 全くそう思わない | Disagree あまりそう思わない | Not sure どちらでもない | Agree まあそう思う | Strongly agree 強くそう思う |
|---|---|---|---|---|---|
| Q11 I consider whether the information is applicable to me<br>自分にもあてはまるかどうか考えた | 1 | 2 | 3 | 4 | 5 |
| Q12 I consider whether the information is credible<br>信頼性に疑問をもった | 1 | 2 | 3 | 4 | 5 |
| Q13 I check whether the information is valid and reliable<br>正しいかどうか聞いたり、調べたりした | 1 | 2 | 3 | 4 | 5 |
| Q14 I collect information to make my healthcare decisions<br>病院や治療法などを自分で決めるために調べた | 1 | 2 | 3 | 4 | 5 |
