# Supplementary material for: Association between health literacy and patient experience of primary care attributes: A cross-sectional study in Japan
Source: PLoS One. 2017 Sep 8;12(9):e0184565. doi: 10.1371/journal.pone.0184565 (PMC5590975; doi:10.1371/journal.pone.0184565)
Supplement: S2 File — (PDF) [file pone.0184565.s002.pdf]

**First contact**	Strongly agree	Somewhat agree	Not sure	Somewhat disagree	Strongly disagree
1. When your Primary Care Practice is closed, is there a phone number you can call when you get sick?	5	4	3	2	1
2. When your Primary Care Practice is closed on Saturday and Sunday and you get sick, would someone from there see you the same day?	5	4	3	2	1
3. When your Primary Care Practice is closed and you get sick during the night, would someone from there see you that night?	5	4	3	2	1
**Longitudinality**	Strongly agree	Somewhat agree	Not sure	Somewhat disagree	Strongly disagree
1. Does your Primary Care Physician (PCP) give you enough time to talk about your worries or problems?	5	4	3	2	1
2. Do you feel comfortable telling your PCP about your worries or problems?	5	4	3	2	1
3. Does your PCP know you very well as a person, rather than as someone with a medical problem?	5	4	3	2	1
4. Does your PCP know what problems are most important to you?	5	4	3	2	1
5. Does your PCP know your complete medical history?	5	4	3	2	1
**Coordination**	Strongly agree	Somewhat agree	Not sure	Somewhat disagree	Strongly disagree
1. Have you ever had a visit to any kind of specialist or special service?		Yes		No/Not sure	
2. Did your PCP suggest you go to the specialist or special service?	5	4	3	2	1
3. Did your PCP discuss with you different places you could have gone to get	5	4	3	2	1

help with that problem?					
4. Did your PCP or someone working with your PCP help you make the appointment for that visit?	5	4	3	2	1
5. Did your PCP write down any information for the specialist about the reason for the visit?	5	4	3	2	1
6. Does your PCP know what the results of the visit were?	5	4	3	2	1
**Comprehensiveness (services available)**<br>For each one, please indicate whether it is available at your PCP's office.	Strongly agree	Somewhat agree	Not sure	Somewhat disagree	Strongly disagree
1. Counseling for mental health problems	5	4	3	2	1
2. Changes in mental or physical abilities that are normal with getting older	5	4	3	2	1
3. Counseling related to dementia	5	4	3	2	1
4. Counseling related to abuse	5	4	3	2	1
5. Counseling related to personal preferences about end of life issues	5	4	3	2	1
**Comprehensiveness (services provided)**<br>In visits to your PCP, are any of the following subjects discussed with you?	Strongly agree	Somewhat agree	Not sure	Somewhat disagree	Strongly disagree
1. Advice about appropriate exercise for you	5	4	3	2	1
2. Advice about regular bowel movements	5	4	3	2	1
3. Advice about over-the-counter medications	5	4	3	2	1
4. Advice about medical information in the media: on TV, in the newspaper, etc.	5	4	3	2	1
5. Advice about a healthy balance of work and rest	5	4	3	2	1
**Community orientation**	Strongly agree	Somewhat agree	Not sure	Somewhat disagree	Strongly disagree
1. Does anyone at your PCP's office ever make home visits?	5	4	3	2	1
2. Does your PCP know about the important health problems of your community?	5	4	3	2	1

3. Does your PCP get opinions and ideas from people that will help to provide better health care?	5	4	3	2	1
4. Does your PCP investigate whether the available health care is meeting the needs of the community?	5	4	3	2	1
5. Does your PCP investigate the concerns people have about health problems in your community?	5	4	3	2	1
